# Co-Occurrence of Child Sexual Abuse and Domestic Violence: A Scoping Review

**DOI:** 10.1177/15248380251383928

**Published:** 2025-11-12

**Authors:** Lenka Olejníková, Molly Dragiewicz, Delanie Woodlock, Michael Salter, Cate Banks

**Affiliations:** 1 Childlight East Asia and Pacific Hub, University of New South Wales, Sydney, Australia; 2 Griffith University, Southport, QLD, Australia; 3 Monash University, Melbourne, VIC, Australia

**Keywords:** child sexual abuse, domestic violence, co-occurrence, polyvictimization, literature review

## Abstract

Research shows that different forms of child maltreatment often co-occur, including child sexual abuse (CSA) and children’s exposure to adult domestic violence (DV). However, the extent of this co-occurrence remains poorly understood. This article presents findings from a scoping review of articles reporting prevalence data for co-occurring CSA and DV. The review included systematic searches and reviews of both scholarly and gray literature. Studies were included if they were published in English and reported childhood experiences of both CSA and DV, with no restrictions on publication date, geographic location, or methodology. This review collates findings from the 19 studies meeting the inclusion criteria. Prevalence rates for co-occurring CSA and DV ranged from 0.9% to 91%. Aside from general methodological differences across the studies, this review identified the target population as an important source of variation in prevalence estimates. Samples drawn from the general, CSA victim/survivor, and DV victim/survivor target populations revealed different patterns, with the CSA population exhibiting significantly higher rates of co-occurrence with DV compared to the other two groups. The review also found that there are sex differences in co-occurring CSA and DV just as there are for each type of abuse individually. This article summarizes the available data on co-occurring CSA and DV, points to areas where future research is needed, and highlights implications for practice.

## Introduction

Recognition of the presence of adult domestic violence (DV) and child sexual abuse (CSA) in the same families is increasing. Many frontline workers, from DV and sexual violence services to health, law, and child protection, now recognize that DV and CSA are often present in the same families and are, in many cases, perpetrated by the same individual. Research confirms this connection, consistently showing that children exposed to one form of abuse are at heightened risk of experiencing other forms (Bidarra et al., 2016; Edleson, 1999; Goddard & Hiller, 1993; Hamby et al., 2010; Haslam et al., 2023; Øverlien, 2010). Despite the growing acknowledgment that abuse is often co-occurring, research that investigates the co-occurrence of CSA and DV remains more limited than that examining either issue in isolation. Due to the dearth of research systematically investigating both issues together, there is a lack of clarity about the extent and nature of the co-occurrence of CSA and DV. We conducted a scoping review to determine what is known about the prevalence of the co-occurrence of CSA and DV to begin to fill this gap and identify areas where further research is required.

### Terminology

We acknowledge the ongoing debates about preferred terminology and definitions of DV, including significant regional, disciplinary, and theoretical variation (see e.g., Myhill & Kelly, 2019). In this article, we define DV as violence or abuse by one current or former intimate partner against another. DV may include, but is not limited to, physical violence, sexual abuse, psychological abuse, technology-facilitated abuse, stalking, coercive and controlling behaviors, and financial abuse. CSA is defined as any contact and non-contact form of sexual behavior against minors. Children cannot legally consent to sexual activity, so all sexual activity by an adult toward a minor is CSA. CSA includes, but is not limited to rape, attempted rape, sexual touching, and taking or sharing sexualized images of children. This definition is consistent with the World Health Organization’s (1999) conceptualization of CSA, which refers to “the involvement of a child (person less than 18 years old) in sexual activity that the child does not fully comprehend, cannot give consent or violates the laws or social taboo of society.” While we use the terms DV and CSA in this article, it is important to note that the studies reviewed use many different terms and definitions. The advantages, shortcomings, and implications of different definitions have been discussed at length elsewhere, and reviewing these is beyond the scope of this article (but see Australian Bureau of Statistics [ABS], 2013; DeKeseredy & Schwartz, 1998; Hamby, 2014; Myhill, 2017; Myhill & Kelly, 2019, for examples). We use specific terms from the individual studies reviewed in the summary table for accuracy.

The overlap between DV and child maltreatment is often referred to as co-occurrence. Co-occurrence is defined as “the fact of two or more things happening or existing at the same time and often in the same place” (Cambridge University Press, n.d.). This review focused on the co-occurrence of CSA and adult DV in order to better understand how these two types of abuse may occur in the same household over the same period of time, and whether research captures instances where both are perpetrated by the same individual. We note that “exposure” to adult DV is more accurate than the older conceptualization of “witnessing” DV, as children in households where adult DV occurs are affected by it in many more ways than just observing or overhearing it. In households were adult DV is present, children often see and hear the abuse, physically or verbally intervene in the abuse, see the aftermath of DV such as injuries, emotional distress, and damage to household items, are enlisted to participate in adult DV by taking part or relaying messages, or are subjected to isolation from friends and family as part of the dynamics of adult DV (Jaffe, 2000; Katz, 2016; Nikupeteri et al., 2021). We emphasize that our interest in co-occurring CSA and DV in this review is distinct from the relationship between CSA victimization and subsequent adult DV victimization or perpetration by the CSA survivor.

### Background

Research on child abuse and DV has largely developed in distinct fields, with a limited number of studies examining both types of abuse in depth (Bidarra et al., 2016). As Edleson (1999) noted 25 years ago, child protection and DV services have different histories. They are underpinned by different policies, funding streams, and theoretical frameworks. While more DV services offer children’s services than in the past and efforts have been made to make child protection systems more aware of DV since the 1990s, referral to the other service area remains a more common model than deep integration of services and systems.

The research is similarly siloed. Most early research on the overlap between child maltreatment and DV drew on research with mothers staying in DV shelters or secondary analysis of administrative data from child protection agencies (Edleson, 1999). The findings of this research were limited for several reasons. First, the data were mainly collected for other purposes or as incidental findings in research focused on other issues (Edleson, 1999).

Second, administrative data are widely recognized as inadequate for understanding the true nature or distribution of child abuse (ABS, 2013; Gillingham, 2020). Administrative data are a by-product of recordkeeping by organizations that come into contact with domestic, family, and sexual violence cases. Agencies such as child protection, specialist DV services, police, courts, family services, corrections, and health care providers collect records that can be analyzed for secondary purposes. These data are free and easy to access as it already exists. However, administrative data are inherently limited because it only captures people who have been identified by systems, elements of phenomena of interest to the organization that collected it, and the information recorded by agency staff (ABS, 2013). Administrative data collection is driven by regulatory and legal requirements rather than understanding the nature of child maltreatment. Substantiation of cases is shaped by gendered and racialized expectations for parents (Henry et al., 2020). In addition, administrative data often differ from self-reports by children in both directions (Brown et al., 1998). Accordingly, administrative data from children’s protective services cannot be taken as providing an accurate representation of the phenomenon of child maltreatment. As Gillingham (2020) argues, “the data held in case files by children’s protective services, at the level of individuals, can provide only a partial and incomplete view of a service user and social problems” (p. 574).

Third, research indicates that parents (DV perpetrators and victims) and children have different perspectives on the abuse, so information collected from adults is inadequate to understand children's experiences (Øverlien, 2010). For example, research involving multiple informants in the same family has found that parents and children’s reports of the presence or absence of abuse often differ, with relative under and over-reporting occurring in varied and unpredictable directions (Sternberg et al., 1998).

Finally, CSA and DV are notoriously difficult to measure individually, with these challenges multiplying when they are examined together. Measurement of CSA and DV are difficult due to disagreements between scholars about pertinent constructs, the ambiguous and context-specific nature of behaviors involved in CSA and DV, and the failure of many studies to discern between abusive and defensive behaviors or playful and intentionally violent acts (see e.g., ABS, 2013; Ackerman, 2016; DeKeseredy & Schwartz, 1998; Hamby, 2014; Myhill, 2017; Myhill & Kelly, 2019; Robinson, 1994). In addition, perpetrators and victims have been shown to justify, deny, minimize, and excuse men’s DV against women in accordance with mainstream heterosexual relationship tropes (Cavanagh et al., 2001; Hearn, 1998; Stamp & Sabourin, 1995; Wood, 2001). Sexual violence may be even more difficult for victims to identify due to persistent cultural norms around men’s rights to sexual access to partners (Basile, 1999; Jeffrey & Barata, 2019) and young children’s limited capacity to understand that sexual acts against them are abusive. Protracted debates about how to best define and measure CSA and DV aside, many participants are required to achieve high-quality statistical analyses. In addition, a large number of survey questions are required to collect basic information about the co-occurrence of CSA and DV, such as the identity of the perpetrator for multiple incidents against multiple people.

## Methods

*How prevalent is the co-occurrence of CSA and DV victimization?* To answer this question, a scoping review was conducted, as it enables to assess the breadth, depth, and nature of the available evidence, highlighting key concepts and research gaps (Munn et al., 2018). Traditional systematic reviews with a systematic appraisal of evidence via a meta-analysis require reviewed studies to be sufficiently similar in design, methodology, and objectives to allow for meaningful synthesis of data (Field & Gillett, 2010). However, the literature that includes data on the prevalence of the co-occurrence of CSA and DV is characterized by considerable heterogeneity, encompassing both qualitative and quantitative studies, a range of sample types and sizes, and inconsistent definitions of CSA and DV. Given this diversity and the limited number of relevant prevalence studies, a scoping review was deemed the most appropriate methodological approach. The review followed the Reporting Items for Systematic Reviews and Meta-Analyses extension for Scoping Reviews (PRISMA-ScR) guidelines (Peters et al., 2020; Tricco et al., 2018), which involved defining eligibility criteria, designing a clear search strategy, specifying the study selection approach, and extracting and charting relevant data.

### Search Strategy and Eligibility Criteria

A systematic search was conducted between August 2023 and December 2024 in two phases using the same search strategy. The full EBSCO and ProQuest databases were searched to identify peer-reviewed publications across relevant disciplines such as public health, sociology, criminology, and social work. Keywords were defined to encompass three key concepts—DV, CSA, and co-occurrence, and were combined using the Boolean operator AND (see Table 1). The inclusion criteria were defined broadly to capture any study published in English at the time of the search that contained data on childhood experiences of both sexual abuse and DV. Studies that examined associations between CSA and DV without reporting any data on prevalence were excluded. Studies employing any research methodology were eligible, including those based on data from adult survivors, children, and their caregivers, or professionals. Studies with both primary and secondary data analyses were included with no restrictions on the publication date or geographical location. Eligible types of publications included peer-reviewed journal articles, books, and PhD theses. Additionally, search of relevant gray literature was also conducted via Google Scholar using the same keywords. Finally, the reference lists of all included studies were reviewed, and any additional publications meeting the inclusion criteria were manually added (see Figure 1).

**Table 1.** Keywords Used for Database Search.

DV Terms	CSA Terms	Co-Occurrence Terms
“intimate partner violence” OR “intimate partner abuse” OR “domestic violence” OR “domestic abuse” OR “intimate violence” OR “battering” OR “family violence” OR “spouse* abuse” OR “gender-based violence” OR “dating abuse” OR batter* OR “marital violence” OR “coercive control”	“child sexual abuse” OR “sexual abuse” OR “incest” OR “child rape” OR “child pornography” OR “child sexual exploitation” OR “child exploitation” OR “child prostitution” OR “juvenile prostitution” OR “child trafficking” OR “child sex trafficking” OR “indecent images”	“co-occurrence” OR “cooccurrence” OR “overlap” OR “crossover” OR “poly-victimization” OR “poly-victimization”

**Figure 1.**
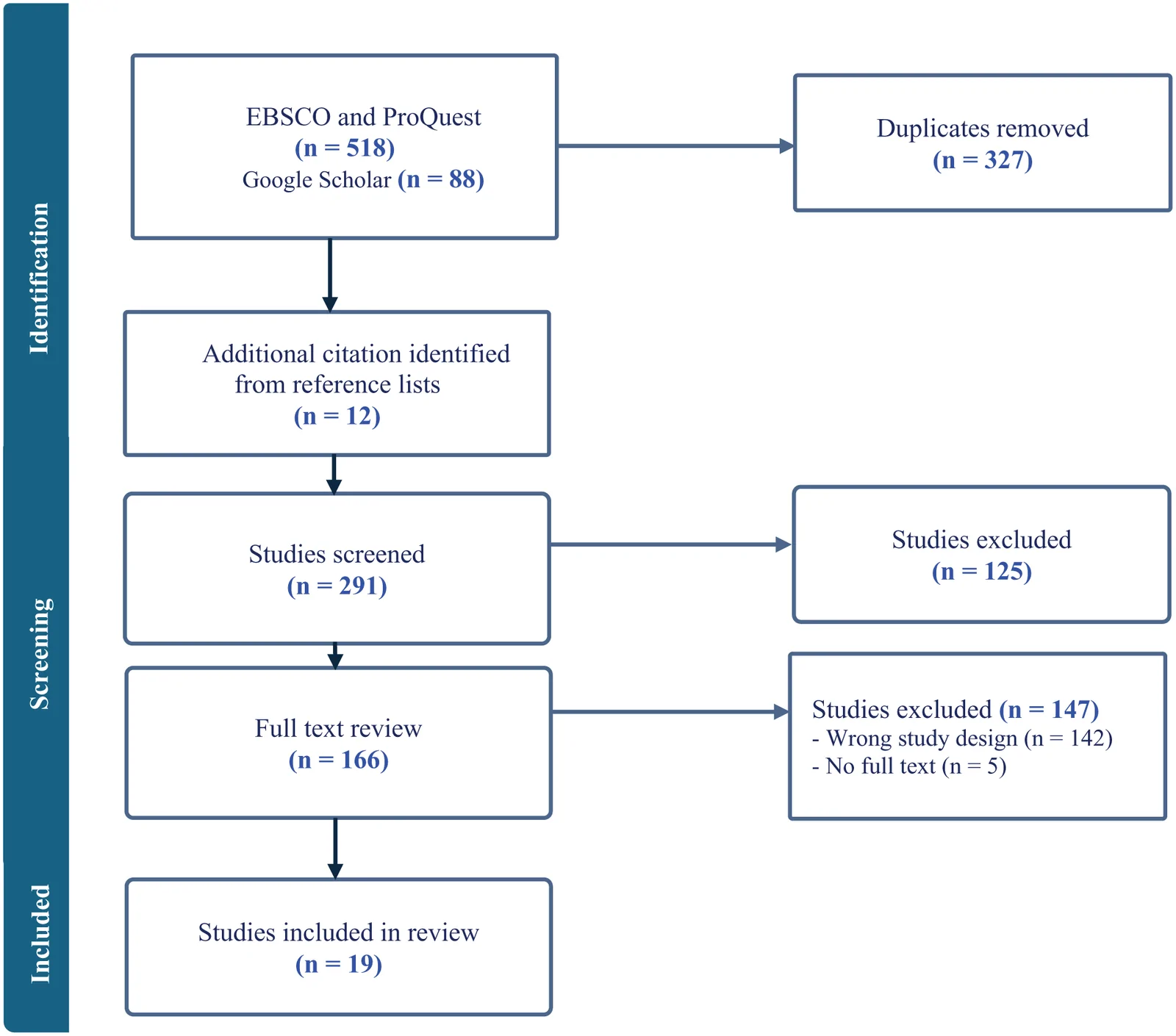
PRISMA flow diagram of the study selection process.

Two reviewers independently searched both EBSCO and SAGE databases, which yielded 291 publications. These publications were then screened by two reviewers in Covidence by applying the eligibility criteria to abstracts. Disagreements were resolved by a third reviewer. Nineteen studies met our inclusion criteria and were included in the final review.

### Charting and Summarizing the Data

The 19 studies were analyzed, and the following information was extracted using the online platform Covidence: (a) publication citation, (b) prevalence rates (including by sex of victim if available), (c) country of data collection, (d) study sample characteristics, (e) methodology, and (f) definitions of CSA and DV. Since only two studies (Hamby et al., 2010; Miranda et al., 2021) reported prevalence estimates for the past 12 months, only lifetime prevalence rates of co-occurrence of CSA and DV in childhood were extracted, as this measure was consistently reported across all included studies. The extracted data were then descriptively analyzed, focusing on the geographic location of the study and methodology. For studies that did not provide overall prevalence estimates but reported separate prevalence rates for female and male victimization along with sample proportions by sex, weighted averages were calculated to provide clearer summary information. In line with PRISMA-ScR guidelines, a critical appraisal of evidence was not conducted.

## Results

The reviewed studies were published between 1986 and 2023, with the majority (*n* = 14, 75%) published after 2000. The studies drew on data from seven different countries: United States of America (*n* = 9), Australia (*n* = 3), United Kingdom (*n* = 3), Canada (*n* = 1), Chile (*n* = 1), China (*n* = 1), and Iraq (*n* = 1). The sample size and type varied significantly across studies, ranging from 11 to 29,801 participants, limiting their comparability. Three earlier studies (16%) had sample sizes of fewer than 50 participants (11–30), eight studies (42%) reported sample sizes ranging from 100 to 1,000 participants (164–730), five studies (26%) included between 1,000 and 10,000 participants (2,064–8,629), and three studies (16%) had samples exceeding 10,000 participants (19,684–29,801). Only two studies were purely qualitative (Bell, 2002; Hooper, 1992) using in-depth interviews and representing the two smallest samples (11 and 15 participants). The remaining 17 studies used quantitative or mixed-methods approaches, drawing on survey (*n* = 11), interview (*n* = 3), or case record (*n* = 3) data. The majority of reviewed studies employed non-representative convenience samples (*n* = 12, 63%), typically with people accessing DV or CSA services. The remaining studies used either representative samples (*n* = 4, 21%) or random samples (*n* = 3, 16%). Consequently, the findings of the majority of reviewed studies have limited generalizability.

### Prevalence of Co-Occurrence of CSA and DV Victimization

The analysis shows that prevalence estimates for co-occurring CSA and DV victimization are characterized by extreme variability (see Table 2). Prevalence estimates ranged from 0.9% (Herbert et al., 2023) to 91% (Bell, 2002). Compared to earlier reviews, we found notable variation in prevalence rates across different target populations.^1^ The studies estimated the prevalence of co-occurring CSA and DV victimization in three different groups:

1. A general population at the country, state, region, or school level. These studies included participants with and without CSA and DV victimization experience and sought to estimate the prevalence of CSA and DV co-occurrence in the population as a whole.
2. Population of DV victims/survivors who were asked about CSA victimization. These studies examined what percentage of identified DV cases included co-occurring CSA.
3. Population of CSA victims/survivors who were asked about childhood DV exposure or victimization. These studies examined what percentage of identified CSA cases included co-occurring adult DV.

**Table 2.** Studies Reporting Prevalence Rates for Co-Occurring CSA and DV (*n* = 19).

Study	Prevalence of CSA and DV	Country	Sample	Methodology
Afifi et al. (2015)	2.8% overall prevalence −2.4% (3.2% female, 1.5% male) experienced sexual abuse, exposure to intimate-partner violence (IPV), and physical abuse −0.4% (0.8% female, 0.04% male,) experienced sexual abuse and exposure to IPV only	Canada (10 provinces)	*n* = 23,395 50% female, 50% male	Secondary data analysis from the Canadian Community Health Survey-Mental Health (CCHS-2012) Representative general population sample
Ahmadabadi et al. (2018)	12% (18.9% female, 1% male) experienced CSA alongside maternal IPV victimization	Australia (Queensland)	*n* = 2,064 59% female, 43% male	Secondary data analysis of the Mater Hospital and University of Queensland Study of Pregnancy (Najman et al., 2005)—a longitudinal study using surveys—mothers asked about IPV at 14-year follow-up and adult children asked about CSA at 30-year follow-up. Convenience sample
Avery et al. (2002)	10.8% of children who were exposed to DV, also experienced CSA	United States	*n* = 570 50% female, 50% male	Secondary data analysis of case records from a DV shelter for women in which children received services. Convenience sample
Bell (2002)	91% of mothers of incestuously abused children reported violence from their partners	United Kingdom (Scotland)	*n* = 11 100% female	In-depth interviews with women from support groups of mothers with incestuously abused children Convenience sample
Bowen (2000)	54% of mothers of sexually abused children reported DV at home	United States (Arizona)	*n* = 402 82% female, 18% male	Survey of families of children seen in a sexual abuse evaluation clinic regarding DV in the child’s home. Children aged from 7 months to 18 years. Convenience sample
Chan et al. (2023)	Mothers who were DV survivors reported that 1% of their children experienced CSA	China (Hong Kong)	*n* = 260	Survey of 260 women living in three major DV shelters about their experience of DV and the experience of child abuse of their children. Convenience sample
Dong (2004)	36.4% of people who witnessed DV in childhood also experienced CSA 22.6% of people who experienced CSA also witnessed DV in childhood	United States (San Diego)	*n* = 8,629 54% female, 46% male	Secondary data analysis of ACE Study (Felitti et al., 1998) that used a survey of adult members of the Kaiser Health Plan who had received an examination at the Health Appraisal Center. Convenience sample
Fuller-Thomson and Agbeyak (2020)	5.3%^a^ (7.7% women, 1.6% men) experienced CSA and exposure to parental IPV (2010) 6.6%^a^ (8.1% women, 4.3% men) experienced CSA and exposure to parental IPV (2012)	United States (7 states)	2010: *n* = 22,868 60% female, 40% male 2012: *n* = 29,801 61% female, 39% male	Secondary data analysis of the Brief Risk Factor Surveillance Survey (BRFSS) of the Centers for Disease Control and Prevention that used phone interviews from a population-based sample of adults with landlines. 2010: DC, Hawaii, Nevada, Vermont, and Wisconsin 2012: Iowa, Tennessee, and Wisconsin. Regionally representative sample
Goddard and Hiller (1993)	40% of CSA victims/survivors reported DV in the family	Australia (Melbourne)	*n* = 206	Secondary analysis based on the data from the Child Protection Unit, Royal Children’s Hospital in Melbourne between January and October 1987. Convenience sample
Hamby et al. (2010)	5.3% of children who witnessed partner violence were also sexually abused by a known adult 70.9% of children who were sexually abused by a known adult also witnessed intimate partner violence	United States	*n* = 4,549 50% female, 50% male	Secondary data analysis of National Survey of Children’s Exposure to Violence (NatSCEV) that used telephone interviews with caregivers of children aged 0–10 and youth 10–17 using random digit dialing. Nationally representative sample
Herbert et al. (2023)	0.9%^a^ (1.3% women, 0.4% men) of people reported both CSA and parental DV 4.3% (6.8% women, 2.1% men) of children exposed to parental DVA reported CSA 30% (26.5% women, 33.3% men) of sexually abused children were exposed to parental DV	United Kingdom (Avon)	*n* = 6,252 52% female, 48% male	Secondary data analysis using Avon Longitudinal Study of Parents and Children (ALSPAC), which invited all pregnant women to participate between 1991 and 1992 in the Bristol area. The data was drawn on reporting from the mothers and their children (Appendix E). Convenience sample
Higgins et al. (2023)	3% (3.9% female, 2.2% male) of people experienced both CSA and exposure to DV in childhood	Australia	*n* = 8,503 50% female, 50% male	Australian Child Maltreatment Study (ACMS) Telephone interviews of people aged 16 and more using random digit dial via an advance text message inviting participation, with a follow-up phone call. Random population sample
Hooper (1992)	81.8% of mothers whose children had been sexually abused by their fathers/father substitutes were abused by the same perpetrator	United Kingdom	*n* = 15 93% female, 7% male	Interviews with 15 women whose children had been sexually abused. Convenience sample
Kellogg and Menard (2003)	52% of sexually abused children reported IPV at home	United States (Texas)	*n* = 164 90% female, 10% male	Analysis of 164 children’s charts from sexual abuse clinic (ages 7–19). Convenience sample
Kennedy et al. (2012)	27% of female adolescents experienced sexual victimization and family violence	United States (Chicago)	*n* = 180 100% female	Survey of urban African American female adolescents 14 to 19 years old at a public charter high school located in a disadvantaged community in Chicago. Convenience sample
McCloskey et al. (1995)	1.8% reported paternal sexual abuse in families with DV (according to children) 3.6% reported paternal sexual abuse in families with DV (according to mothers)	United States	*n* = 730 (365 mothers and 365 children—50% female, 50% male)	In-depth interviews of battered and nonbattered women and their children. Convenience sample
Miranda et al. (2021)	11.6% of adolescents experienced sexual assault by a known adult and IPV exposure	Chile	*n* = 19,684 50% female, 50% male	Survey of students from 7th to 11th grade in 699 schools using stratified sampling in three stages (schools, classrooms, and students). Random sample
Saed et al. (2013)	1.6% experienced both CSA and IPV exposure 8.1% of those who were exposed to IPV also experienced CSA	Iraq (Erbil)	*n* = 300 66% female, 34% male	Survey of college students from four colleges (Education, Art, Nursing, and Medicine) in Erbil. Random sample
Truesdell et al. (1986)	73% of mothers of incestuously abused children reported physical violence from their spouse	United States (Texas)	*n* = 30 100% female	Survey of a mother’s group of the incest treatment program offered by the Texas Department of Human Resources. Convenience sample

*Note*. CSA = child sexual abuse; DV = domestic violence.

a If data on overall prevalence were not reported in a study, weighted averages were calculated using reported estimates for female and male victimization and the proportion of women and men in the sample.

Grouping prevalence rates by target population reduces the variability and reveals a noticeable pattern among the different groups. As Table 3 shows, studies that draw on general population target populations report the lowest average prevalence rates for CSA and DV co-occurrence, ranging from 0.9% (Herbert et al., 2023) to 27% (Kennedy et al., 2012) with a mean of 7.9%. The study by Kennedy et al. (2012) stands out with a 27% prevalence estimate, however, it is the only study in this group that included only female African American adolescents from a low-income area. Each of these demographic categories is associated with an increased risk of both CSA and DV victimization (see Assink et al., 2019; Black et al., 2001; Giano et al., 2020). When this study is excluded, the average prevalence across the general population studies drops to 5.5%. The second group of studies that assessed the prevalence of CSA victimization in identified DV victim/survivor populations reported slightly higher rates on average, ranging from 1% (Chan et al., 2023) to 36.4% (Dong, 2004) with a mean of 9.8%. The highest prevalence estimates for co-occurring CSA and DV were identified in research with identified CSA victim/survivor populations, ranging from 22.6% (Dong, 2004) to 91% (Bell, 2002) with a mean of 57.3%. Figure 2 provides a graphical representation of the data distribution within and across the groups using comparative distribution dot plot.2 However, considerable differences in research design across studies, such as sampling, type of data, and the definitions of CSA and DV used in each study, may also contribute to the observed variability in prevalence rates both within and across the three population groups.

**Table 3.** Prevalence of Co-Occurring CSA and DV Victimization by Target Population.

General Population			DV Population			CSA Population		
Study	%	*n*	Study	%	*n*	Study	%	*n*
Herbert et al. (2023)	0.9	6,252	Chan et al. (2023)	1.0	260	Dong (2004)	22.6	8,629
Saed et al. (2013)	1.6	300	McCloskey et al. (1995)	2.7	730	Herbert et al. (2023)	30.0	6,252
Afifi et al. (2015)	2.8	23,395	Hamby et al. (2010)	5.3	4,549	Goddard and Hiller (1993)	40.0	206
Higgins et al. (2023)	3.0	8,503	Herbert et al. (2023)	4.3	6,252	Kellogg and Menard (2003)	52.0	164
Fuller-Thomson and Agbeyaka (2020)	5.3	52,669	Saed et al. (2013)	8.1	300	Bowen (2000)	54.0	402
Fuller-Thomson and Agbeyaka (2020)	6.6	52,669	Avery et al. (2002)	10.8	570	Hamby et al. (2010)	70.9	4,549
Miranda et al. (2021)	11.6	19,684	Dong (2004)	36.4	8,629	Truesdell et al. (1986)	73.0	30
Ahmadabadi et al. (2018)	12.0	2,064	—	—	—	Hooper (1992)	81.8	15
Kennedy et al. (2012)	27.0	180	—	—	—	Bell (2002)	91.0	11

*Note*. CSA = child sexual abuse; DV = domestic violence.

**Figure 2.**
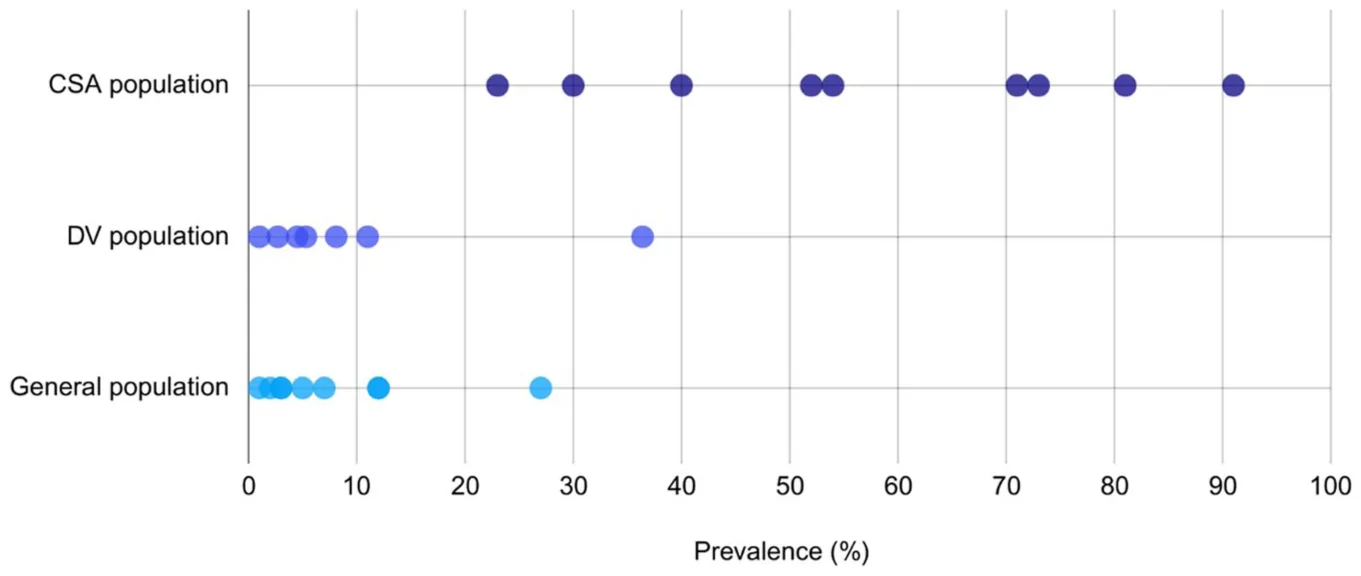
Visualization of prevalence of co-occurring child sexual abuse and domestic violence victimization according to target population.

#### Sampling

Sample size appears to be one contributing factor, with smaller sample sizes generally associated with higher prevalence rates. Studies reporting prevalence rates below 10% (*n* = 12) had the largest mean sample size of 8,662 participants, whereas those reporting rates between 10% and 50% (*n* = 8) had a smaller mean sample size of 5,777 participants. In contrast, studies with prevalence rates above 50% (*n* = 6) had the smallest mean sample size, averaging just 862 participants. The three studies with the smallest samples, comprising 11 participants (Bell, 2002), 15 participants (Hooper, 1992), and 30 participants (Truesdell et al., 1986), reported the highest prevalence rates of 91%, 81.8%, and 73%, respectively. Similarly, sample type may also influence prevalence estimates. Studies using random or representative samples reported rates ranging from 1.6% to 70.9%, with a mean of 14%, whereas studies based on convenience samples showed a broader range (0.9%–91%) and a higher mean prevalence of 31.9%. Clinical convenience samples, in particular, may yield higher co-occurrence rates, as they often include individuals with more severe or repeated histories of abuse and are therefore more likely to report multiple forms of maltreatment. Nearly all studies reporting prevalence rates above 50% were based on small clinical samples from CSA services. However, Hamby et al. (2010), using a large, nationally representative sample of 4,549 participants, reported a 70.9% co-occurrence rate in CSA population, comparable to rates found in much smaller clinical samples. These patterns suggest that while high prevalence rates are unlikely to be primarily attributable to sampling methods alone, both sample size and sample type likely contribute to the variability in prevalence estimates observed across and within the three population groups.

#### Data Sources

The studies included in our review generally used three different approaches to data collection for identifying the co-occurrence of CSA and DV: self-reports from victims/survivors, reports from children’s caregivers, and administrative data. Most studies (*n* = 14) collected information directly from victims/survivors via surveys or interviews. In terms of CSA victimization, data were obtained through self-reports from children (Miranda et al., 2021), from both children and adult survivors (Higgins et al., 2023; Kennedy et al., 2012), or adult survivors only (Afifi et al., 2015; Ahmadabadi et al., 2018; Dong, 2004; Fuller-Thomson & Agbeyaka, 2020; Saed et al., 2013). Some studies supplemented or combined victim/survivor reports with information from caregivers, typically mothers (Goddard & Hiller, 1993; Hamby et al., 2010; Herbert et al., 2023; McCloskey et al., 1995), and three older studies (Bell, 2002; Hooper, 1992; Truesdell et al., 1986) relied exclusively on maternal reports. Additionally, three studies used administrative data from victim/survivor service records (Avery et al., 2002; Bowen, 2000; Kellogg & Menard, 2003).

Similarly, children’s exposure to DV at home was primarily determined through self-reported data. These sources included surveys and interviews with mothers about their own experiences (Ahmadabadi et al., 2018; Bell, 2002; Bowen, 2000; Chan et al., 2023; Hooper, 1992; Truesdell et al., 1986), as well as with children (Miranda et al., 2021) or adult survivors (Afifi et al., 2015; Dong, 2004; Fuller-Thomson & Agbeyaka, 2020; Higgins et al., 2023; Kennedy et al., 2012; Saed et al., 2013) about witnessing or experiencing DV in the household. Some studies collected reports from both children and mothers (McCloskey et al., 1995), from children and/or their caregivers (Goddard & Hiller, 1993; Hamby et al., 2010), or combined reports from adult survivors and their mothers (Herbert et al., 2023). Only two studies relied exclusively on case records from service providers (Avery et al., 2002; Kellogg & Menard, 2003).

No consistent patterns were identified between the type of data source used and the variation in prevalence estimates. Nevertheless, the choice of data source may influence findings in individual studies, particularly in relation to the disclosure of CSA victimization, thereby contributing to the broader variability in estimates. Self-reported data on child maltreatment, particularly when collected retrospectively, are prone to recall inaccuracies. Disclosure by children may be hindered by feelings of shame, fear of disbelief, or concerns regarding potential consequences for the victim or the perpetrator (Lemaigre et al., 2017). Adult survivors may also underreport such experiences due to a lack of recognition of the incident as abuse or as a result of emotional responses such as confusion and shame (Langeland et al., 2015). Furthermore, individuals subjected to abuse in early childhood may have limited or no memory of the event due to their developmental stage at the time (London et al., 2008). Self-reported data may therefore underestimate the true prevalence of maltreatment; however, they are generally considered to capture more accurate rates than official records (Afifi et al., 2015).

On the other hand, parents or caregivers may provide an incomplete account of the child’s experience of maltreatment, either due to a lack of awareness of the abuse or intentional minimization (Finkelhor et al., 2005; Øverlien, 2010). Mothers may be reluctant to report the sexual abuse of their children to service providers due to fears of losing custody. Research indicates that maternal allegations of child abuse by fathers, particularly in cases involving physical or sexual abuse, are associated with an increased risk of custody loss (Meier, 2020). Finally, secondary analysis of records collected for other purposes has serious limitations including missing or miscategorized data as workers prioritize their bureaucratic needs, have limited time, and may rely on anecdotal disclosures rather than direct questioning (Gillingham, 2020). Fear of custody loss may help explain why Chan et al. (2023) reported a co-occurrence rate of only 1% in DV populations, which is the lowest prevalence rate observed within this group and the only study relying solely on mothers’ self-reports in a DV shelter.

#### Definitions

Most studies (*n* = 10) defined CSA as contact sexual abuse, typically encompassing multiple types of abuse (e.g., sexual touching, attempted rape, and rape). Notably, Fuller-Thomson and Agbeyaka (2020) is the only study that defines CSA solely as rape. Five other studies used a broader definition of CSA that incorporated contact and non-contact sexual abuse, such as flashing/sexual exposure, verbal sexual harassment, voyeurism, and recording or showing sexually explicit material (Ahmadabadi et al., 2018; Chan et al., 2023; Higgins et al., 2023; Miranda et al., 2021; Truesdell et al., 1986).^3^ DV was predominantly narrowly defined as physical violence (*n* = 10), while seven studies used broader definitions comprising verbal, emotional, financial, or sexual abuse (Ahmadabadi et al., 2018; Bell, 2002; Chan et al., 2023; Higgins et al., 2023; Hooper, 1992; McCloskey et al., 1995; Truesdell et al., 1986).^4^

Differences in definitions have long been known to affect prevalence findings, with broader definitions capturing a larger portion of abuse yet increasing the risk of mischaracterizing innocuous behaviors as abuse (Ackerman, 2016; DeKeseredy & Schwartz, 1998). In the present review, however, definitional differences did not appear to significantly affect co-occurrence estimates. Among studies using a narrow definition of CSA, limited to contact abuse, prevalence rates ranged from 0.9% (Herbert et al., 2023) to 81.8% (Hooper, 1992), with a mean of 21.2%. Similarly, studies employing broader definitions of CSA reported rates ranging from 1% (Chan et al., 2023) to 73% (Truesdell et al., 1986), with a comparable mean of 20.1%. In contrast, definitional differences in DV appeared to have a more noticeable impact. Studies using narrower definitions of DV reported co-occurrence rates ranging from 0.9% (Herbert et al., 2023) to 70.9% (Hamby et al., 2010), with a mean of 21.2%. Those employing broader DV definitions showed a wider range, from 1% (Chan et al., 2023) to 91% (Bell, 2002), and a higher average prevalence of 33.4%. It is important to note that studies in this review used various combinations of narrow and broad definitions for CSA and DV, which may explain why we did not observe a more pronounced impact of different definitions on the co-occurrence estimates.

#### Sex Differences

Our review identified a pattern of sex differences in the prevalence of co-occurring CSA and DV victimization. On average, girls experience co-occurring CSA and DV at a higher rate than boys (see Figure 3). However, this observation is based only on the studies (*n* = 5) that disaggregated the prevalence rates for co-occurring CSA and DV by sex. All five of these studies were in the group studying general target populations (see Table 4).

**Figure 3.**
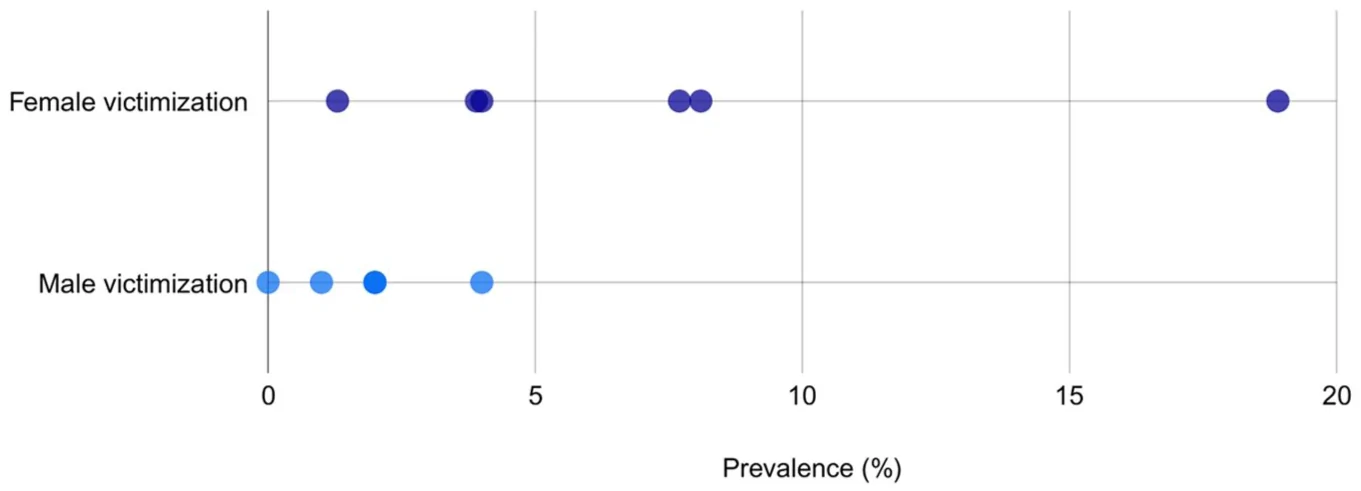
Visualization of co-occurring child sexual abuse and domestic violence victimization in general target populations disaggregated by sex (*n* = 6).

**Table 4.** Co-Occurring CSA and DV Victimization in General Target Populations Disaggregated by Sex (*n* = 6).

Study	Female Victimization (%)	Male Victimization (%)
Afifi et al. (2015)	4.0	1.5
Ahmadabadi et al. (2018)	18.9	1.0
Fuller-Thomson and Agbeyaka (2020, 2010 data)	7.7	1.6
Fuller-Thomson and Agbeyaka (2020, 2012 data)	8.1	4.3
Herbert et al. (2023)	1.3	0.4
Higgins et al. (2023)	3.9	2.2

## Discussion

This scoping review aimed to clarify what is known about the co-occurrence of CSA and DV, whether the same individual perpetrated CSA and DV, and how frequently this overlap in perpetrator identity was reported in existing studies. Our findings reveal key patterns in how this relationship has been studied, as well as significant gaps in evidence and methodology. One key observation from this review is the patterned variation in prevalence rates in different target populations. Studies focused on CSA victim populations were associated with a high likelihood that DV was also present. Studies focused on DV victim populations were less likely to report CSA was present. Another key finding is sex differences in rates of DV and CSA co-occurrence, with girls experiencing more co-occurring CSA and DV than boys. Nonetheless, the majority of studies failed to report disaggregated data on sex. A third observation is the lack of essential information about the identity of the perpetrators of CSA and DV in the reviewed studies, which limits our understanding of whether the abuses co-occurred (i.e., occurred in the same household and/or were committed by the same person).

### Prevalence Rates Across Different Target Populations

Our review of the research on the co-occurrence of CSA and DV found that if CSA is present, DV is highly likely to be present, with an average co-occurrence rate of 57.3%. This finding has important implications for CSA and child safety organizations and workers, indicating that universal screening for adult DV should be undertaken when CSA is detected, a longstanding suggestion by previous researchers (Kellogg & Menard, 2003). Given the limited integration between these fields, it appears that it would be beneficial to allocate more resources to bring DV into the purview of CSA organizations. While DV is associated with increased risk of CSA with a mean co-occurrence rate of 9.8%, the relationship is clearly weaker than the converse. As such, DV organizations should continue to screen for child maltreatment. However, these differences suggest different etiologies for or typologies of CSA and DV that require further investigation to reveal these dynamics.

### Sex Differences

Our review identified a pattern of sex differences in the prevalence of co-occurring CSA and DV victimization, with girls more likely to experience co-occurring CSA and DV than boys. Sex differences in rates of CSA (Dunne et al., 2003; Mathews et al., 2023) and DV (Cunningham & Anderson, 2023; Mathews et al., 2025) victimization and perpetration are well documented. Nonetheless, 14 of 19 of the studies included in this review did not provide prevalence rates for co-occurring CSA and DV disaggregated by sex. All five of the studies that presented this information were in the group studying general target populations (see Table 4). While sex differences are known to reflect biological as well as gendered (social and structural) contributing factors to health inequality, the studies captured in this review did not provide information about social and structural factors contributing to the observed sex differences (Fishman et al., 1999; Kaufman et al., 2023). Identifying the gendered factors shaping observed sex differences is essential for prevention as these can inform sex-specific prevention campaigns that can address the different risk profiles for girls and boys.

### Co-Occurrence or Poly-Victimization/Multi-Type Maltreatment?

Our review found that information about perpetrator identity was limited in studies discussing the co-occurrence of CSA and DV. Only four of the 19 studies clearly stated whether CSA and DV involved the same or different perpetrators, or whether the abuses were concurrent. Notably, no study has reported this level of detail since 2003. The remaining 15 studies did not provide sufficient information to determine who perpetrated the CSA, or whether the abuse occurred alongside DV.

Among the four studies that provided perpetrator data, the following patterns were observed:

1. Intimate partners of the mother as dual perpetrators of CSA and DV

All four studies identified the mother’s partner (husband, ex-husband, or cohabitee) as both the perpetrator of DV and CSA:

• Hooper (1992) reported that in 11 of 15 cases where children were sexually abused, the abuser was the mother’s partner.
• Bell (2002) found that 10 of 11 mothers of incestuously abused children had experienced partner violence.
• Kellogg and Menard (2003) observed that 58% of in-home male child sexual offenders also physically abused the child’s mother.

McCloskey et al. (1995) reported that paternal sexual abuse occurred in 3.6% of families with DV, with mothers suspecting additional incest in 6% of cases.

2. In-home male perpetrators (including biological fathers) as both physically and sexually abusive
• Kellogg and Menard (2003) also found that half of the in-home males who were physically violent toward children also sexually abused them, reinforcing the pattern of multi-type maltreatment within the same perpetrator.
3. Extrafamilial and multiple perpetrators in the context of DV
• McCloskey et al. (1995) reported that 25% of children in families with DV had been sexually abused outside the home, highlighting that children exposed to DV were also at risk of abuse by extrafamilial perpetrators.
4. Other relatives and extended family members
• Hooper (1992) additionally identified four cases where the abuser was a relative other than the mother’s partner, including the woman’s father and son, and two of the partner’s relatives (e.g., the child’s uncle or cousin).

Fifteen of the 19 studies reviewed did not specify who perpetrated the CSA or whether it overlapped in time with DV. These studies focused primarily on experiencing multiple different types of violence or abuse in childhood, usually referred to as polyvictimization (Finkelhor et al., 2007) or multi-type maltreatment (Haslam et al., 2023), and the effects of childhood maltreatment, rather than examining the relational dynamics of abuse. The absence of perpetrator identity obscures our understanding of the distinctions between polyvictimization and co-occurrence.

### Limitations

Our scoping review was subject to several limitations. First, it was limited to English-language publications, which may have led to the omission of relevant findings from non-English academic literature. Second, as discussed above, most studies reviewed did not report information about the identities of the perpetrators. As a result, we could not determine whether children were subjected to sexual abuse and DV by the same perpetrator, which limits the strength of our conclusions regarding the co-occurrence of CSA and DV. Lastly, it is important to emphasize that, unlike systematic reviews, scoping reviews are not designed to rigorously assess the validity of individual study findings and to produce cumulative estimates. Therefore, our findings should be interpreted with caution, especially the mean co-occurrence rates of CSA and DV presented for each target population. They are intended to provide a general summary of the literature rather than credible prevalence estimates.

In addition to the limitations, this review highlights the complexities of estimating the co-occurrence of CSA and DV that stem from the nature of CSA and DV, the challenges involved in studying each issue individually and understanding their relationship to one another. Debates about the prevalence of both types of abuse and how to best quantify them are well-documented and ongoing, underpinned by theoretical disagreements among scholars. Challenges related to the experience of CSA and DV also shape research. CSA and DV are often clandestine crimes. Many of the behaviors involved are deeply context-specific, meaning that additional questions are required beyond items about what behaviors occurred. Self-reporting is affected by victims’/survivors’ internalized feelings of shame and ability to recognize abuse when it occurs. As a result, CSA and DV are regularly under-identified, including by victims themselves. In sum, CSA and DV prevalence are difficult to measure well. These measurement difficulties are magnified when two types of abuse are considered concurrently.

### Implications for Practice

This review of the extant research points to important directions for practice. Our review affirms earlier recommendations for organizations and individuals who come into contact with CSA cases to universally screen for DV and increase CSA workforce capability around DV (Hester, 2011; Kellogg & Menard, 2003). While such recommendations have been made for decades, they remain pertinent (Mandel, 2024). This review also confirms marked sex differences in co-occurring CSA and DV. Accordingly, risk assessments based on risks to one sex cannot be assumed to accurately apply to the other. This review indicates that some of the most nuanced research on the co-occurrence of CSA and DV was conducted more than two decades ago by scholars interested in incestuous abuse and gender and power in families using qualitative methods and small samples. This approach has been overtaken by quantitative and epidemiological approaches that have made significant progress in documenting the harms of co-occurring DV and child maltreatment. However, basic questions about who the perpetrators of co-occurring CSA and DV are and the dynamics and timing of the abuse remain unanswered.

### Directions for Future Research

This review has clarified gaps in knowledge about the co-occurrence of CSA and DV to guide future research. First, despite growing recognition of the correlation between CSA and DV, the nature, dynamics, or direction of this relationship remain poorly understood. Second, future prevalence studies geared toward understanding the co-occurrence of CSA and DV in the general population and victim/survivor target populations should collect and report information about the identity of the perpetrators of both types of abuse and whether the abuse was concurrent or not. Third, studies should employ larger, more representative samples to avoid artificially inflated rates of CSA and DV victimization. They should also adopt broader, contextualized definitions of both forms of abuse to capture the full range of experiences, consistent with current understandings of what constitutes CSA (Greijer & Doek, 2025) and DV (Postmus et al., 2018). Fourth, given the clear pattern of sex differences in co-occurring CSA and DV, researchers should report disaggregated data on victim and perpetrator sex for these types of abuse. In addition, research on the social and structural factors contributing to the sex differences observed in this study is needed (Kaufman et al., 2023). Fifth, given the complexity of these cases, qualitative and quantitative research with victim/survivor target populations is necessary to flesh out our understanding of how CSA and DV are correlated. Typologies of each type of abuse may be helpful to inform targeted prevention and intervention campaigns. Research focused on girls and boys is necessary to understand each group’s experiences, gendered risk factors, and the factors shaping outcomes for survivors.

## Conclusion

This scoping review aimed to determine what the research tells us about the prevalence of the co-occurrence of CSA and DV victimization. The 19 studies reviewed reported a wide range of prevalence rates, from 0.9% to 91%, with notable patterns across different target populations. First, where CSA is identified, adult DV is highly likely to also be present. Second, while CSA rates are, on average, somewhat higher in families where DV is present compared to the general population, this association appears significantly weaker than the reverse (DV is more consistently present in cases where CSA has been identified). Third, in studies that disaggregated data by sex, women and girls were found to be at greater risk of experiencing co-occurring CSA and DV. Finally, although co-occurrence is not the norm, it affects a sizable minority of children, particularly those who experience CSA. These findings suggest that the co-occurrence of CSA and DV merits greater attention in both research and practice.

**Table:** Critical Findings of the Review.

Prevalence	CSA victims/survivors experience the highest levels of CSA and DV co-occurrence with prevalence ranging from 22.6% to 91%. In contrast, prevalence is markedly lower among the general population (0.9%–27%) and the DV-affected population (1%–36.4%). The intra-group variability can be largely attributed to methodological differences.
Sex differences	CSA and DV co-occurrence is higher among females (1.3%–18.9%) compared to males (0.4%–3%).
Terminology	Definitional differences of CSA and DV across the studies affect who counts as victim/survivor, impacting the prevalence rates. The lack of standardized definitions also prevents comparability of the studies.
Co-occurrence	Studies generally do not identify perpetrators of CSA and DV, making it impossible to determine whether the two types of abuse occurred concurrently within the same family unit.

*Note*. CSA = child sexual abuse; DV = domestic violence.

**Table:** Implications of the Review for Practice, Policy, and Research.

DV screening should be part of every CSA case investigation.
The CSA workforce should be trained in the area of DV.
Co-occurrence of CSA and DV is a gendered phenomenon, which should be reflected in approaches to risk assessment.
The nature and dynamics of the co-occurrence of CSA and childhood DV should be further studied to enable design of effective policy and intervention programs.
Research on CSA and DV should include the identities of perpetrators to facilitate assessment of true co-occurrence as distinct from polyvictimization.
Research should use larger, representative samples and broader definitions of CSA and DV to ensure more accurate prevalence estimates.
Prevalence of CSA and DV co-occurrence should be reported disaggregated by sex.

*Note*. CSA = child sexual abuse; DV = domestic violence.

## Notes

1. A target population is defined as the group that has the characteristics that an analysis aims to investigate. The target population may be broad, like the general population of a state or all students at a university, or it may be more specific, like identified DV victims/survivors or identified CSA victims/survivors (see e.g., Stuart et al. 2018; Willie, 2023). In this article, we discuss the prevalence rates for the target populations to which each reported estimate speaks (general populations that include victims and non-victims, identified CSA victims, and identified DV victims), whether that is the focus of the full study from which they are taken or not.

2. Each dot in the graph represents an estimate from Table “Critical Findings of the Review.”

3. Four of the earlier studies do not explicitly define child sexual abuse (Avery et al., 2002; Bell, 2002; Bowen, 2000; Goddard & Hiller, 1993).

4. Two of the earlier studies do not explicitly define DV (Avery et al., 2002; Goddard & Hiller, 1993).
